# Supplementary material for: Practical behavioural solutions to COVID-19: Changing the role of behavioural science in crises
Source: PLoS One. 2022 Oct 12;17(10):e0272994. doi: 10.1371/journal.pone.0272994 (PMC9555670; doi:10.1371/journal.pone.0272994)
Supplement: S1 Appendix — The translated flyer (originally Dutch) that was distributed to inform people of the upcoming experiment in the supermarket. (PDF) [file pone.0272994.s001.pdf]

Are you participating? We investigate how we can facilitate physical distancing, to make it easier for all of us.

In March, the Smart Distance Lab will be conducting research in this supermarket to find out how we can facilitate physical distancing. On various days we will be using different aids, such as traffic lights, arrows, and directional signs – to see what works. Your participation is a small effort, but of great importance: together we will learn how we can keep our distance more easily, to make the supermarkets safer. With this knowledge we can open other public spaces in a safer way.

How can you participate?

1. **Carrying a remote sensor.** When you visit the supermarket, you will be asked to carry a sensor with you. At the end of your visit, you will hand the sensor back in. The sensor measures the distance to other sensors in the supermarket, which allows us to see whether it is possible for people to keep their distance. The sensor is completely anonymous and will not be linked to you as a customer in any way. Participation is of course completely voluntary.

2. **We would like to hear what you think of corona.** You can also use a button to indicate how you experienced your visit. On some days there will be interviewers who want to do a short interview about corona. Of course, participation is again completely voluntary.

When does the reserach take place?

Wednesday March 17th until Saturday March 20th; and Wednesday March 24th until Saturday March 27th.

Who is leading this research?

This research is a collaboration of the University of Amsterdam, Smart Distance Lab, Ministry of Economic Affairs and Climate Change, Games for Health, ITOM, and the PLUS.

Results

After the measurements have been completed, we will inform you of the most important results of the research by means of a second flyer. This way you will bet he first to know what works to facilitate physical distancing in the supermarket.

Questions and contact

For questions, ideas, and suggestions, please contact info@smartdistancelab.nl. During the survey, staff from the Smart Distance Lab will be at the entrance to answer your questions.

If you have any comments or remarks about the study, please contact the researchers responsible: Tessa Blanken, t.f.blanken@uva.nl en Charlotte Tanis, c.c.tanis@uva.nl.
